# The 1897 Meeting-place of the U. S. V. M. A.

**Published:** 1896-10

**Authors:** 


### EDITORIAL.

"1897." The meeting of 1897 of the U. S. V. M. A. will be of such vital importance to every member of the profession in America that its place of convening should be well considered by one and all. With such a strong staff of officers as will direct affairs during the ensuing year there should be better results follow the remarkable meeting at Buffalo. It is these thoughts and delightful anticipations that prompt us at this time to call attention to the all-important necessity of wisely determining the best place for the Convention of 1897. The one great question for each and all to consider is, In what city can the highest and best interests of the profession be promoted? And to this question every other thought and pleasant recollection must be made subservient. Where will the opportunities be greatest for the largest number to assemble? Where should we go to strengthen and sustain the weakest arm of our profession in this country? These are the questions to be determined, and one and all should think twice, and think seriously, before placing with the Secretary conclusions that must guide and enable the Executive Committee to decide this all-important question of—Detroit, Cleveland, Columbus, Kansas City, or Nashville! Which?

